# Perfect and broadband acoustic absorption by critically coupled sub-wavelength resonators

**DOI:** 10.1038/srep19519

**Published:** 2016-01-19

**Authors:** V. Romero-García, G. Theocharis, O. Richoux, A. Merkel, V. Tournat, V. Pagneux

**Affiliations:** 1 LUNAM Université, Université du Maine, CNRS, LAUM UMR 6613, Av. O. Messiaen, 72085 Le Mans, France

## Abstract

Perfect absorption is an interdisciplinary topic with a large number of applications, the challenge of which consists of broadening its inherently narrow frequency-band performance. We experimentally and analytically report perfect and broadband absorption for audible sound, by the mechanism of critical coupling, with a sub-wavelength multi-resonant scatterer (SMRS) made of a plate-resonator/closed waveguide structure. In order to introduce the role of the key parameters, we first present the case of a single resonant scatterer (SRS) made of a Helmholtz resonator/closed waveguide structure. In both cases the controlled balance between the energy leakage of the several resonances and the inherent losses of the system leads to perfect absorption peaks. In the case of the SMRS we show that systems with large inherent losses can be critically coupled using resonances with large leakage. In particular, we show that in the SMRS system, with a thickness of *λ*/12 and diameter of *λ*/7, several perfect absorption peaks overlap to produce absorption bigger than 93% for frequencies that extend over a factor of 2 in audible frequencies. The reported concepts and methodology provide guidelines for the design of broadband perfect absorbers which could contribute to solve the major issue of noise reduction.

The balance between the leakage rate of energy out of a resonant scatterer and its inherent losses has been shown of fundamental relevance for its transmission and reflection properties^1^,^2^,^3^,^4^. When the leakage and the inherent losses are well balanced, the critical coupling condition is fulfilled, and then, a perfect destructive interference between the transmitted and the internal fields leads to maximum absorption at the resonance frequency^1^,^5^. The critical coupling also paved the way to produce perfect absorption (PA) of the incoming waves. In particular, the coherent perfect absorption^6^, being the time-reversed counterpart to laser emission^7^, makes use of the critical coupling mechanism to perfectly absorb the two port coherent excitation in a lossy resonant medium.

PA is of particular interest for many applications such as energy conversion^8^,^9^, time-reversal technology^10^,^11^, coherent perfect absorbers^6^,^7^ or soundproofing^12^,^13^ among others. In optics, several configurations using Bragg reflectors^14^, Fabry-Pérot cavities with metamaterial mirrors^15^, layered media with Kerr nonlinearity^16^ or graphene-based hyperbolic metamaterials^17^ have been recently used to obtain nearly PA of the incident radiation by critical coupling. Moreover PA has been obtained in plasmonic planar structures via the critical coupling of the surface plasmon modes^18^, while this has been also used for heat generation in plasmonic metamaterials^19^. In microwaves, metamaterial resonators that couple separately electric and magnetic fields show PA within a single unit layer^20^. In acoustics, coherent perfect absorption has been studied numerically by controlling the coherence of the input waves in a two port system showing a dynamically tuned absorption coefficient on the target material up to unity^21^. Recently, impedance matched membranes^13^ and bubble metascreens^22^ have been used to turn acoustic reflectors into perfect absorbers.

These advances in PA have motivated an increasing interest on the design of perfect absorbers which at the same time are sub-wavelength structures^23^. It is desirable to avoid bulky structures and reach a broadband and angular performance. In acoustics, the majority of the sub-wavelength absorbers works efficiently only within a limited narrow band at low frequencies^13^. The broadband performance of the perfect absorption at low frequencies using sub-wavelength structures remains a great scientific challenge.

In this work, we experimentally and theoretically show a perfect and broadband absorption using a sub-wavelength multi-resonant scatterer (SMRS) made of a viscoelastic porous plate in-line loaded to a closed waveguide. Here, perfect and broadband absorption is possible by the critical coupling of two adjacent and highly leaky hybridized resonances of the total system made of the SMRS and the closed cavity behind. In our case, the characteristics of these resonances (resonance frequency and leakage rate) are tuned by varying the length of the backing cavity. For given configurations, it is possible to balance the high inherent losses with the strong leakage of two adjacent hybridized resonances and this leads to perfect and broadband absorption. In particular, this is successfully realized by (*a*) using a multi-resonant element (resonant plate) which is (*b*) highly lossy (viscoelastic porous) and (*c*) by controlling the interplay between the energy leakage of the resonances (hybridized resonances) into the waveguide and the inherent losses of the system (PA by engineered critical coupling). Making use of these three features and overlapping several perfect absorption peaks, we observe an absorption larger than 93% for broad band that extend over a factor of 2 in audible frequencies. The resulting structure presents a sub-wavelength thickness of *λ*/12 with a diameter of *λ*/7.

From the above mentioned features, the most important is the latter one, the PA by engineered critical coupling. Thus in order to reveal its importance we will first consider a single resonant scatterer (SRS) with small inherent losses made of a Helmholtz resonator side-loaded to the waveguide, rigidly backed. After that, the relevance of the effects of both the inherent losses and the multiple hybridized resonances on the absorption performance of the SMRS will be described.

## Results

### Samples

**Figure 1:**
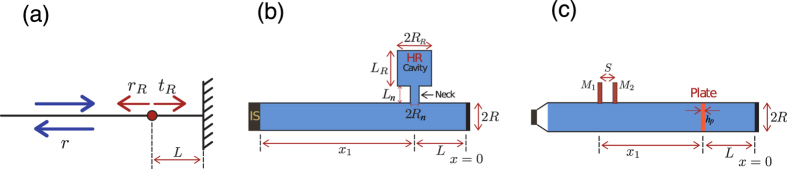
(**a**) Asymmetric Fabry-Pérot resonator made of a resonant element (red point) and a rigid backing at distance *L* from the resonator. (**b**,**c**) respectively show the SRS and the SMRS set-ups. In the set-up (**b**) an Impedance Sensor (IS)^25^ is used for the measurements. In (**c**) two microphones placed before the sample are used to experimentally characterize the system^29^.

The configurations analyzed in this work can be considered as an asymmetric Fabry-Pérot cavity of length *L* with two different mirrors, i.e., the resonant scatterer, considered as a point-scatterer because it is sub-wavelength, and the rigid backing [as schematically shown in Fig. 1(a)]. The frequency range considered here is well below the first cut-off frequency of the higher propagative modes in the waveguide, therefore the problem is considered as one-dimensional. The absorption of this system can be expressed as *α* = 1 − |*r*|^2^, where *r* is the complex reflection coefficient obtained from the standard three-medium layer Fresnel equation^24^,


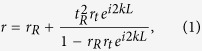


where the time harmonic convention is *e*^−*iωt*^, *r*_*R*_ and *r*_*t*_ are the reflection coefficients of the resonant element and of the termination, respectively (in our case, *r*_*t*_ = 1), and *k* = *ω*/*c* is the wave number in the cavity of length *L* with *ω* = 2*πf* the angular frequency with *f* the frequency. Considering inherent losses in this configuration, the PA is fulfilled when the reflection coefficient is zero, i.e., when the superposition of the multiple reflections in the cavity [second term on the right hand of Eq. (1)] destructively interferes with the direct reflection from the resonant element [first term on the right hand of Eq. (1)].

Figure 1(b) shows the set-up used for the SRS with the Helmholtz resonator side-loaded to the closed waveguide. The Helmholtz resonator is composed of a neck of length *L*_*n*_ = 2 cm with radius *R*_*n*_ = 1 cm, a cavity with tunable length, *L*_*R*_, and radius *R*_*R*_ = 2.15 cm. The waveguide has a radius *R* = 2.5 cm and *L* = 15 cm. The viscothermal losses at the walls of the waveguide and of the resonator are characterized by both a complex wave vector and a complex impedance^25^,^26^.

The set-up with the in-line loaded viscoelastic porous plate is shown in Fig. 1(c). The viscoelastic porous plate is made of polyurethane, in particular of Methylene diphenil diisocyanate (MDI). This material is used to produce polyurethane rigid foams for high performance insulation applications. The plate has a thickness of *h*_*p*_ = 3.5 mm and a radius of *R*_*p*_ = 2.2 cm. It is clamped in a tube of *R* = 2.2 cm. The viscoelastic material of the plate is characterized by a Poisson’s ratio of *ν* = 0.1, with a complex Young’s modulus *E* = *E*_0_ + *iηω* (*E*_0_ = 220 kPa and *η* = 7 Pa s) and a complex mass density of form 
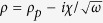
 (*ρ*_*p*_ = 28 kg/m^3^ and *χ* = 350 kg m^3^ s^1/2^), *η* and *χ* are describing the viscoelastic losses of the plate and have been extracted by acoustic measurements (see Supplementary Materials).

### PA by engineered critical coupling

We start by the analysis of the SRS geometry [see Fig. 1(b)] in order to show how we engineer the critical coupling condition in our systems to obtain PA. Theoretically, we will also reveal the importance of the total amount of inherent losses.

**Figure 2:**
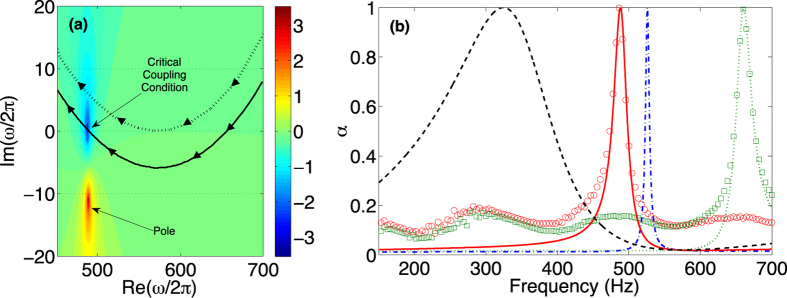
SRS configuration. (**a**) Represents the complex frequency map of log |*r*| for the SRS with *L*_*R*_ = 8.3 cm. Black dashed (continuous) line represents the trajectory of the zero of |*r*| for the lossless (lossy) case in the complex plane increasing *L*_*R*_ [sense of the increasing shown by arrows over the lines]. (**b**) Red continuous and green dotted lines (open red circles and open green squares) represent the absorption coefficient *α* for the configurations (*L*_*R*_, *f*_*CC*_) = (8.3 cm, 484.5 Hz) and (*L*_*R*_, *f*_*CC*_) = (3.9 cm, 647 Hz). Blue dash-dotted line represents the absorption coefficient for the configuration (*L*_*R*_, *f*_*CC*_) = (7 cm, 526 Hz) with half inherent losses of the experimental case. Black dashed line represents the absorption coefficient for the configuration (*L*_*R*_, *f*_*CC*_) = (16 cm, 330 Hz) with 20 times the inherent losses of the experimental case.

By changing *L*_*R*_ from 0 to 15 cm, i.e., by changing the resonant frequency of the Helmholtz resonator, we study the trajectory of the zero of the reflection coefficient in the complex frequency plane (see Methods) for the lossless case. We notice that in this case the zero appears at a complex frequency the real part of which is related to the resonance frequency while the imaginary part is related to the leakage rate of the system. Moreover, in the lossless case, the zero never crosses the real frequency axis as it was expected. The reflection coefficient remains equal to one, |*r*| = 1, for all frequencies on the real frequency axis. Figure 2(a) (black dashed line) shows the trajectory of the zero of the reflection coefficient that is produced by a hybridized resonance due to the interaction between the resonance of the Helmholtz resonator and the resonance of the backing cavity. As shown by the arrows over the trajectory, the zero moves to lower real frequencies as *L*_*R*_ increases. Moreover, if the zero is close to the real frequency axis, the leakage of the resonance is very small and it is a quasi-trapped mode^27^. Then the characteristics of the resonances, i.e., the resonant frequency and the leakage rate are related to the real and imaginary part of the zero in the lossless complex frequency plane respectively (see Supplementary Material).

Now we consider the viscothermal losses in the system and we observe that the trajectory of the zero down-shifts (black continuous line) with respect to the lossless case. The critical coupling condition is satisfied at the frequency *f*_*CC*_ at which the trajectory of the zero crosses the real frequency axis. For the analyzed system, one can clearly see two crossing points, i.e., two different configurations producing PA. These points correspond to (*L*_*R*_, *f*_*CC*_) = (8.3 cm, 484.5 Hz) and (*L*_*R*_, *f*_*CC*_) = (3.9 cm, 647 Hz). Color map in Figure 2(a) shows the reflection coefficient [Eq. (1)] in the complex frequency plane for the configuration (*L*_*R*_, *f*_*CC*_) = (8.3 cm, 484.5 Hz). Similarly, a complex map for the configuration (*L*_*R*_, *f*_*CC*_) = (3.9 cm, 647 Hz) with the zero in the real frequency axis can also be obtained (see Supplementary Materials). We find analytically PA (*α* = 1) for the two above mentioned configurations at 484.5 Hz and 647 Hz as shown in Fig. 2(b), in agreement with the crossing points of the trajectory of the zero with the real frequency axis represented in Fig. 2(a). Experiments show also very good agreement with the theoretical predictions, producing 100% of absorption for these configurations at the corresponding frequencies with a relative narrow bandwidth of frequencies due to the small leakage of the resonance (the zero in the lossless case is close to the real axis).

Generally, by changing the inherent losses of the system, we can move the trajectory of the zero in the complex plane, and we can always find a configuration with the good balance between the energy leakage and the inherent losses of the whole resonator to fulfill the critical coupling condition and activate the PA. In particular, increasing of inherent losses in the system produces two main effects: the critical coupling condition is shifted in frequencies and the PA peak becomes broadband because the critical coupled resonances are more leaky.

In order to show these effects, we theoretically analyze the cases of weak and large inherent losses in the SRS configuration. Dash-dotted blue line and dashed black line in Fig. 2(b) represent the absorption coefficient for the configurations (*L*_*R*_, *f*_*CC*_) = (7 cm, 526 Hz) and (*L*_*R*_, *f*_*CC*_) = (16 cm, 330 Hz) each one with the right amount of inherent losses to accomplish the critical coupling. The first (second) one corresponds to a situation with 0.5 (20) times the inherent losses of the experimental case. With small amount of inherent losses, one can find a very narrow PA peak while with large amount of inherent losses the PA peak becomes broad. The broad character is due to the large energy leakage of the critically coupled resonance (see Supplementary Materials).

### Perfect and Broadband Absorption: Highly lossy SMRS

The study will now be focused on the system with the SMRS [Fig. 1(c)]. In this case, the resonant element is a viscoelastic porous plate coupled to a closed cavity. The plate presents two flexural resonances in the analyzed frequency range and introduces high inherent losses into the system (see Supplementary Material).

**Figure 3:**
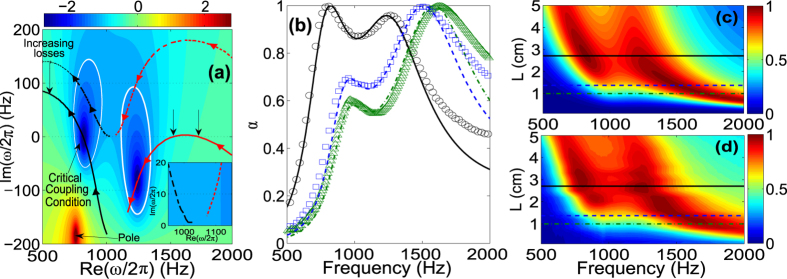
SMRS configuration. (**a**) Represents the complex frequency map of log |*r*| for the SMRS with *L* = 2.71 cm. Black dashed (Continuous) and red dashed (continuous) lines represent the trajectory of the zero of |*r*| for the first and second resonances for the lossless (lossy) case as *L* is increased (sense of the increasing shown by arrows over the lines). White continuous lines show the isoline *α* = 0.9. Inset shows a zoom of the complex frequency map. In (**b**) green dot-dashed (green open triangles), blue dashed (open blue squares) and black continuous (black open circles) lines show the analytical (experimental) absorption coefficients for the cases *L* = 1 cm, *L* = 1.37 cm and *L* = 2.71 cm [horizontal lines in (**c**,**d**)] respectively. (**c**,**d**) show the theoretical and experimental maps for the dependence of *α* on both the length of the cavity and the frequency.

In Fig. 3(a), we analyze the trajectories of the zeros of Eq. (1) for both cases, the lossless and the lossy (with the inherent losses corresponding to the experimental analyzed system), as we change the cavity length *L* from 0 to 13 cm. For each case, there are two trajectories corresponding to the two zeros of the reflection coefficient coming from the two hybridized resonances of the SMRS. As shown by the arrows over the trajectories, the two zeros move to lower real frequencies as the cavity length *L* increases and its imaginary part can also be tuned, i.e., the resonance frequency and the leakage of the hybridized resonances can be controlled by the geometry of the system, in our case by the cavity length *L*.

As soon as the inherent losses are taken into account, the trajectories of the zeros of the lossless case are down-shifted. Here, as a major difference with respect to the SRS case previously discussed, the zeros that are down-shifted by the presence of inherent losses illustrate modes with larger leakage rate. Therefore, highly leaky modes will reach the critical coupling condition. In this case we expect broad absorption peaks. We notice that for very small values of *L*, the zero of the first resonance is close to the real frequency axis, then small amount of losses will be enough to critically couple these quasi-trapped modes. This fact has been exploited recently to show that, with small amount of inherent losses, this kind of systems can be used to design sub-wavelength impedance matched systems^13^ in a very narrow range of frequencies. In contrast, in the current work, we analyze the multi-resonant case in which the inherent losses are substantially large, and the hybridized resonances have a large leakage rate to produce broad PA peaks that can overlap, generating broadband absorption.

In Fig. 3(a), we can observe that the trajectory of the zero of the first resonance crosses the real frequency axis for the configuration (*L*, *f*_*CC*_) = (2.71 cm, 820 Hz). Moreover, the trajectory of the zero of the second resonance crosses the real frequency axis in two points, for the configurations (*L*, *f*_*CC*_) = (1.37 cm, 1508 Hz) and (*L*, *f*_*CC*_) = (1 cm, 1703 Hz). The color map in Fig. 3(a) represents the reflection coefficient [Eq. (1)] in the complex frequency plane for the configuration (*L*, *f*_*CC*_) = (2.71 cm, 820 Hz), the color maps for the configurations representing the two other crossing points can be found in the Supplementary Material. We find analytically PA (*α* = 1) for the three above mentioned configurations at 820 Hz, 1508 Hz and 1703 Hz as shown in Fig. 3(b). Experimental results show also very good agreement with the theoretical predictions, producing 100% of absorption for these configurations at the corresponding frequencies. In contrast to the case of SRS, the PA peaks shown here are broad in frequencies because the critically coupled resonances have large leakage rate.

Interestingly, for the configuration (*L*, *f*_*CC*_) = (2.71 cm, 820 Hz), we can observe in Fig. 3(b) that the PA peak overlaps with a nearly PA peak produced by the second resonance. While the zero of the reflection coefficient of the first resonance is on the real frequency axis producing the PA, the zero of the second resonance is off-axis but it has a big influence on the absorption coefficient (*α* = 0.96). In order to understand the mechanism that produces this overlap, first we analyze the dependence of *α* on both *L* and *f*. Figure 3(c,d), respectively show the theoretical and experimental results. As the cavity length changes, here we clearly see that both the resonant frequency and the leakage rate of the resonance of the system can be tuned. Particularly, we can find a zone (around the configuration with *L* = 2.71 cm) where the two modes are close to one another with high absorption values. This means that in this region, the resonances present similar leakage rates (imaginary part of the zero in the complex frequency plane) that almost compensate the high inherent losses for the two resonances. Specially, for the configuration with *L* = 2.71 cm, the first resonance has the right leakage rate to perfectly compensate the high inherent losses (critical coupling condition), while the second resonance is close to this compensation. Then, our system present two highly leaky modes relatively close that can be tuned with the freedom to change the length cavity *L* together with a material of the plate that has the right amount of losses to critically couple these hybridized and leaky resonance.

Finally, we quantify the absorption produced by the overlapping mechanism in our system. We have evaluated the isoline *α* = 0.9 in the complex frequency plane [white contours in Fig. 3(a)]. The isolines *α* = 0.9 around the zeros of the first and second resonances, cross the real frequency axis, denoting a substantial increase of the absorption in this frequency range. The absorption is bigger than 93% for frequencies that extend over a factor of 2 in audible frequencies from 700 Hz to 1400 Hz. The dimensions of the SMRS are deep sub-wavelength both in thickness, *λ*/12, and in diameter, *λ*/7. It is worth noting here that we have performed a parametric analysis of the dependence of the absorption coefficient on the other geometrical parameters of the plate (see Supplementary Material). The geometry results optimal in terms of broadband and perfect absorption.

### Impedance matching

A perfect absorber consists of a lossy material that absorbs the incoming waves with an input impedance that is perfectly matched to the impedance of the surrounding medium in order to avoid the reflection. As recently shown, perfect absorption can be easily achieved if the thickness of the material is large enough^28^. Using the critical coupling condition, we have designed small and broadband perfect absorber, minimizing the thickness of the absorber and, reaching a sub-wavelength structure. Here, we evaluate the acoustic impedance of such systems in order to demonstrate that the previous designs are impedance matched.

**Figure 4:**
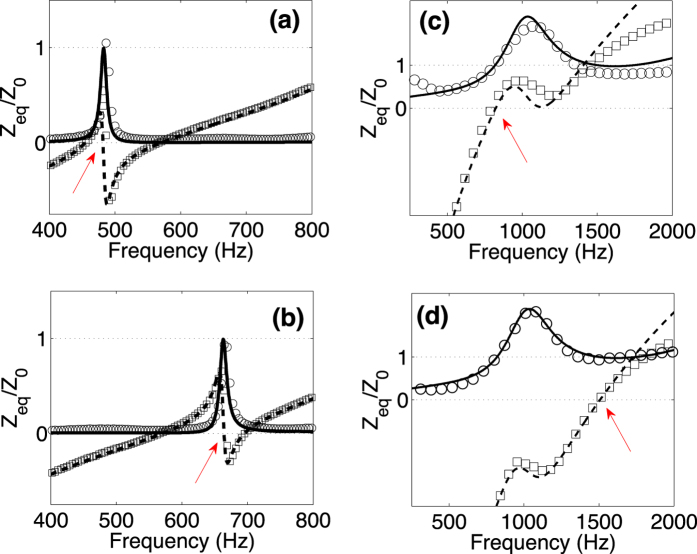
Normalized acoustic impedances. Continuous (Open circles) and dashed (open squares) lines represent the real and the imaginary part of the theoretical (experimental) normalized acoustic impedance for (**a**) SRS configuration with *L*_*R*_ = 8.3 cm, (**b**) SRS with *L*_*R*_ = 3.9 cm, (**c**) SMRS resonant configuration with *L* = 2.71 cm and (**d**) SMRS with *L* = 1.37 cm. Red arrows points the critical coupling condition for each case in agreement with Figs 2 and 3.

Figure 4(a,b) show the theoretical and experimental results of the normalized acoustic impedances of two different perfect absorbers made of the SRS. Figure 4(c,d) show the theoretical and experimental results of the normalized acoustic impedances of two perfect sub-wavelength absorber made of the SMRS. Comparing the acoustic impedance shown in Fig. 4 with Figs 2 and 3 we can see that at the frequencies where the PA is achieved, the impedance matching condition is fulfilled.

## Discussion

**Figure 5:**
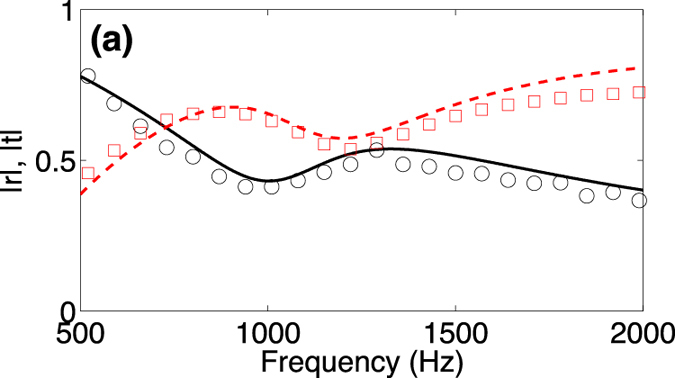
(**a**) Magnitude of the reflection and transmission coefficients of the viscoelastic porous plate. Red continuous dashed (Red open squares) and black continuous line (black open circles) corresponds to the theoretical (experimental) values of the reflection and transmission coefficient respectively.

We start the discussion by the analysis of the reflection, *r*, and the transmission, *t*, coefficients of the viscoelastic porous plate used in this work. Figure 5 shows the theoretical and the experimental values of these coefficients. We observe that their magnitudes are oscillating around 0.5 over the frequency range of interest.

PA can be also obtained using the concept of the coherent perfect absorption (CPA) by controlling the coherence of the input waves on the two sides of the 1D resonator^6^. Recently, Wei *et al.*^21^ have extended the concept of CPA into the domain of acoustics. They have shown the conditions to obtain symmetric and antisymmetric CPA using lossy resonant elements. To exhibit CPA with symmetrical inputs, the resonant element should accomplish the condition *r* = −*t* = 0.5, while for having PA with anti-symmetrical inputs, the resonant element should accomplish the condition *r* = *t* = 0.5. If we regard the values of the reflection and transmission coefficient of the viscoelastic plate used in this work, we observe that the viscoelastic porous plate is close to the conditions to generate anti-symmetric CPA.

In this work, we place the viscoelastic porous plate in front of and close to a rigid backing, forming the SMRS. Therefore, the role of the rigid backing is to introduce the right amount of anti-symmetric input into the system to generate the PA. The direct reflection from the viscoelastic plate brings a *π* shift, whereas the reflection from rigid end has a zero phase. As a consequence, as the length of the cavity is much smaller than the wavelength, we can consider that the phase shift due to propagation into the cavity is negligible. Therefore at the exit of the SMRS, a destructive interference between the two paths produces the PA. An analogue discussion can be done for PA in the SRS configuration but considering symmetrical CPA conditions, i.e. the rigid backing in the SRS systems introduces the right amount of symmetric input to activate the PA.

The methodology developed in this work, based on the analysis of reflection coefficient in the complex frequency plane, provides a general procedure to obtain the critical coupling condition to activate the PA in closed systems. It is worth noting that analytical conditions can be found for the case of high *Q* factors^1^,^5^,^6^, but for the case of resonators with large inherent losses (which is the case for most of the acoustic resonators) other tools are needed to identify the critical coupling condition. In this work we have shown that the analysis of the zeros of the reflection coefficient in the complex frequency plane provides a general tool to design perfect absorbers.

We have exploited the critical coupling to experimentally and analytically show perfect and broadband absorption of acoustic waves with sub-wavelength resonators. We have shown here that, making use of the inherent losses of the system, one can overlap PA and nearly PA peaks to produce broadband PA absorption with a sub-wavelength structure with thickness of *λ*/12 and diameter of *λ*/7 made of a viscoelastic porous plate in-line loaded to a closed waveguide. The controlled interplay between the energy leakage of the several resonances and the inherent losses of this system reveals perfect absorption peaks that can overlap to produce absorption bigger than 93% for frequencies that extend over a factor of 2 in audible frequencies. This could contribute to solve the major issue of noise reduction at low frequencies and, due to the fact that the phenomenology shown here is based on generic wave principles, studies in different domains of wave physics can also be motivated.

## Methods

### Experimental set-ups

The experimental set-up used for the characterization of the SRS is composed of an impedance sensor within which there are two microphones and a piezoelectric source (see details in ref. 25) and the sample (a rigid end cylindrical tube, side loaded with the Helmholtz resonator). The sample is composed of a cylindrical waveguide with an inner radius *R* = 2.5 cm and a wall thickness of 0.5 cm. The length correction of the neck due to radiation was experimentally measured to be 1.04 cm by comparing the input impedances (experimentally and theoretically) for different volumes of the Helmholtz resonator cavity (see details in ref. 25). The system made by the Helmholtz resonator and the cavity is obtained by transferring the measured input impedance by the impedance sensor to the position of the Helmholtz resonator which 80 cm far from the source.

The experimental set-up used for the SMRS is based on the ISO-10534-2 for the determination of sound absorption coefficient and impedance in impedance tubes^29^. We use an impedance tube of 2 cm of radius. The front tube has a loud speaker at one end to generate a plane wave. Two microphones are installed in the front tube to measure the incident and reflected waves in order to characterize both the reflection amplitude and phase. The absorption coefficient is obtained as *α* = 1 − |*r*|^2^, with *r* being the measured reflection coefficient.

### Complex frequency plane

For the case of weakly lossy resonators, the *Q* factor that characterizes its total losses can be regarded as 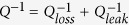 and calculated as *Q*^−1^ = Δ*f*_*res*_/*f*_*res*_, where *f*_*res*_ is the resonant frequency and Δ*f*_*res*_ is the width at −3 dB. Under these perturbative conditions, it is known that pronounced resonant dips with PA will be excited when the critical coupling condition, i.e., *Q*_*leak*_ = *Q*_*loss*_, is fulfilled in an rigid backed waveguide^1^.

However, once the resonators are quite lossy, i.e. out of this perturbative regime, the approximated expressions previously discussed are not valid and different tools have to be used to identify the critical coupling condition. In this work we use a graphical representation of the scattering function in the complex frequency plane, which is a classical method in scattering theory and gives a general overview of the physical behavior in the system. We analyze the reflection coefficient *r*, which corresponds to the scattering function of the systems analyzed in this work, evaluated in the complex frequency plane, i.e., substituting *ω* = *ω*_*R*_ + *iω*_*I*_ in *k*. Generally, in the lossless case, one finds pairs of poles (at complex frequency *ω*_*P*_) and zeros (at complex frequency 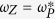) of *r* symmetrically distributed around the real frequency axis^27^. The zeros (poles) are in the positive (negative) half imaginary frequency plane (see Supplementary Materials). When the inherent losses are introduced into the system, the zeros and the poles are in general down-shifted. In the case where the zero is on the real frequency axis (*ω*_*Z*_ = *ω*_*R*_ + *i*0 = 2*πf*), *r*(*ω*_*Z*_) = 0 so the critical coupling condition is fulfilled and the PA is obtained.

### Theory

In acoustics the sound pressure *P*, and normal acoustic volume velocity *U*, on the two faces of a sample that is composed of *N* elements are related by the following expression,


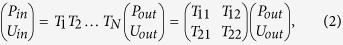


where *T*_*i*_ is the transmission matrix of the *i*-th element. Namely, the total transmission matrix of the sample is given by the product of the transmission matrices of each element. Our configurations are made by three different elements: In-line elements (resonant plates), parallel elements (Helmholtz resonators), and cavities of length *L* composed of a waveguide of cross-section area *S*. The transmission matrices of these three elements are


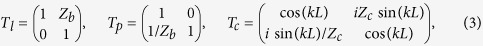


where *l*, *p* and *c* are the subindex for the in-line, parallel and cavity elements; 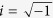, *Z*_*b*_ is the frequency dependent impedance of the resonant element, *Z*_*c*_ and *k* are the complex impedance and wave number considering the viscothermal losses.

## Additional Information

**How to cite this article**: Romero-García, V. *et al.* Perfect and broadband acoustic absorption by critically coupled sub-wavelength resonators. *Sci. Rep.* **6**, 19519; doi: 10.1038/srep19519 (2016).

## Supplementary Material

Supplementary Information
