# Supplementary material for: Perfect and broadband acoustic absorption by critically coupled sub-wavelength resonators
Source: Sci Rep. 2016 Jan 19;6:19519. doi: 10.1038/srep19519 (PMC4726070; doi:10.1038/srep19519)
Supplement: Supplementary Information [file srep19519-s1.pdf]

Supplementary material:
Perfect and broadband acoustic absorption by critically coupled sub-wavelength resonators

V. Romero-García[1], G. Theocharis[1], O. Richoux[1], A. Merkel[1],
V. Tournat[1] and V. Pagneux[1]

[1]LUNAM Université, Université du Maine, CNRS, LAUM UMR 6613, Av. O. Messiaen, 72085 Le Mans, France

I. CHARACTERIZATION OF THE RESONANT PLATE

In order to characterize the acoustic response of the viscoelastic porous plate, we experimentally evaluate its transmission matrix using the method of Song and Bolton[1]. Four microphones are used to acquire the pressure field. Two after and two before the resonant plate embedded in a waveguide. A loudspeaker is used to generate a plane wave. Using the measured pressure and velocity at the four positions and considering symmetry and reciprocity conditions, the elements of the transmission matrix can be obtained[1,2].

Figure 1 shows the experimental results (open symbols) and the theoretical predictions (lines) of the four elements of the transmission matrix of the resonant plate. According to the expression of T_l (see Methods), Fig. 1(a) shows the elements T_{11}, T_{22} which are equal due to the symmetry condition. Figure 1(b) shows the element T_{21} which is zero because of the continuity of the velocity, and (c) shows the element T_{12} which corresponds to the impedance of the resonant element.

The acoustic impedance of the circular plate of radius R_p, Z_b is given by[3]

$$Z_b = -\frac{\imath\omega m}{\pi R^2}\frac{I_1(k_pR_p)J_0(k_pR_p) + J_1(k_pR_p)I_0(k_pR_p)}{I_1(k_pR_p)J_2(k_pR_p) - J_1(k_pR_p)I_2(k_pR_p)}, \tag{1}$$

where J_n and I_n are the regular and modified Bessel's functions of the first kind of order n, $D = Eh_p^3/12(1-\nu^2)$ is the flexural rigidity, being h_p the thickness of the plate and ν the Poisson's ratio. $k_p^2 = \omega\sqrt{\rho h_p/D}$ is the wave number in the plate and $m = \rho h_p \pi R_p^2$ is the mass

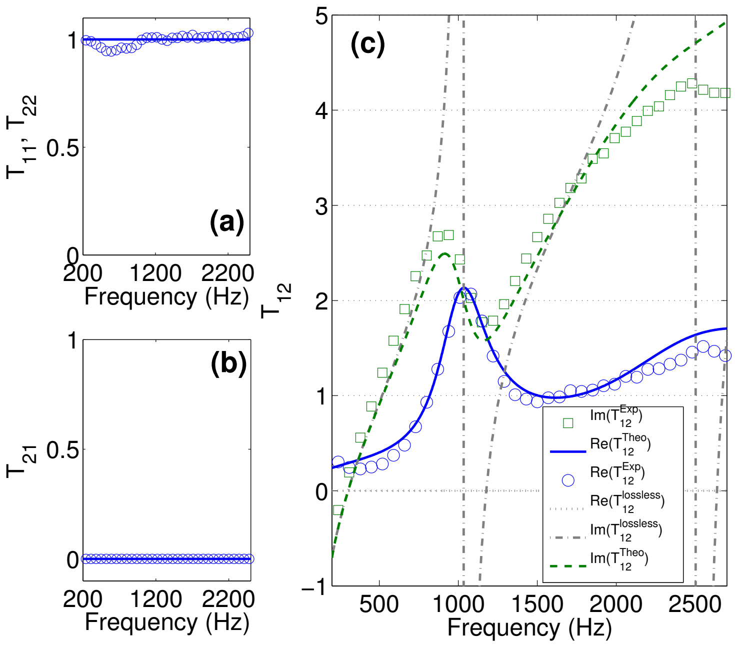

FIG. 1. (Color online) Characterization of the transmission matrix of the viscoelastic plate: Theoretical and experimental evaluation of its elements. (a) Continuous line (open circles) shows the theoretical (experimental) values for T_{11} ($= T_{22}$ for symmetry conditions). (b) Continuous line (open circles) shows the theoretical (experimental) values for T_{21}. (c) Continuous and dashed lines (open circles and squares) show the theoretical (experimental) values for the real and imaginary parts of T_{12} (acoustic impedance of the viscoelastic plate Z_b) respectively. As an eye guide we show the values for the acoustic impedance of a perfect lossless plate with the grey dotted (real part) and dash-dotted lines (imaginary part).

of the plate. The plate analyzed experimentally has $R_p = 2.2$ cm, $h_p = 3.5$ and $\nu = 0.1$. Viscoelastic and viscothermal losses are considered in this resonant plate by considering a complex Young's modulus with a frequency dependent imaginary part, $E = E_0 + \imath\eta\omega$ ($E_0 = 220$ kPa and $\eta = 7$ Pa s) and a complex mass density of form $\rho = \rho_p - \imath\chi/\sqrt{\omega}$ ($\rho_p = 28$ kg/m^3).

Figure 1(c) shows experimental values of the real and imaginary parts of the impedance of the resonant plate (open symbols), and the analytical values calculated from Eq. (1) considering $\chi = 350$ kg m^3 s$^{1/2}$ and $\eta = 7$ Pa s (continuous line). The agreement between experiments and theory is fairly good. This result is an implicit validation of the modelling of the considered inherent losses. As an eye guide we also represent the lossless impedance

of the plate ($\chi = 0$ kg m^3 s$^{1/2}$ and $\eta = 0$ Pa s). The points with $\mathrm{Im}(Z_b) = 0$ correspond to resonances of the plate. For the lossless case, $\mathrm{Im}(Z_b) = \infty$ correspond to the anti-resonance of the plate. Notice that the presence of inherent losses, increases the real part of the impedance and the imaginary part is smoothed.

II. REFLECTION COEFFICIENT IN THE COMPLEX FREQUENCY PLANE

The reflection and transmission coefficients of the resonant element are obtained in this work by means of the transfer matrix method. Then, they are introduced in the Eq. (1) of the manuscript to evaluate the reflection coefficient of the complete system. By considering the frequency to be complex, we can evaluate the reflection coefficient of the whole system, i.e., resonant element and backing cavity, in the complex frequency plane. For our single port configuration the scattering matrix is a 1×1 matrix, and it is equal to the reflection coefficient.

In order to emphasize the properties of reflection coefficient in the complex frequency plane, we present in more details the case of the parallel resonant configuration made of a Helmholtz resonator (HR) and a backing cavity ($L = 15$ cm) as shown in Fig. 1(b) of the manuscript. In particular, Figs. 2(a)-(c) show the complex maps of the reflection coefficient for the lossless case of the configurations with $L_R = 10$, 8.3 and 7 cm respectively. By time reversal symmetry, a pole of the reflection coefficient (scattering matrix) is associated to a zero[4]. The pair zero and pole are placed respectively in the positive and negative half space (the temporal harmonic dependence of the type $e^{-\imath\omega t}$). In the lossless case, they are symmetric with respect to the real frequency axis at which the value of the reflection coefficient is $|r| = 1$. One can also observe in Figs. 2(a)-(c) the change of the shape of the reflection coefficient around its zero as the zero approaches to the real axis. The closer to the real axis the zero is, the sharper the shape of the reflection coefficient around zero is. This shape characteristic, in the lossless case, is related with the leakage of the resonant system. The zeros close to the real frequency axis represent quasi-trapped modes[4].

In order to characterize the configurations with high absorption we define the isoline $\alpha = 0.9$, enclosing configurations with absorption $\alpha \geq 0.9$ in the complex frequency plane. In Figs. 2(a)-(c) we plot this isoline for the analyzed cases. Due to the fact that we study the lossless case, both the zero and the isoline never cross the real frequency axis, so the PA

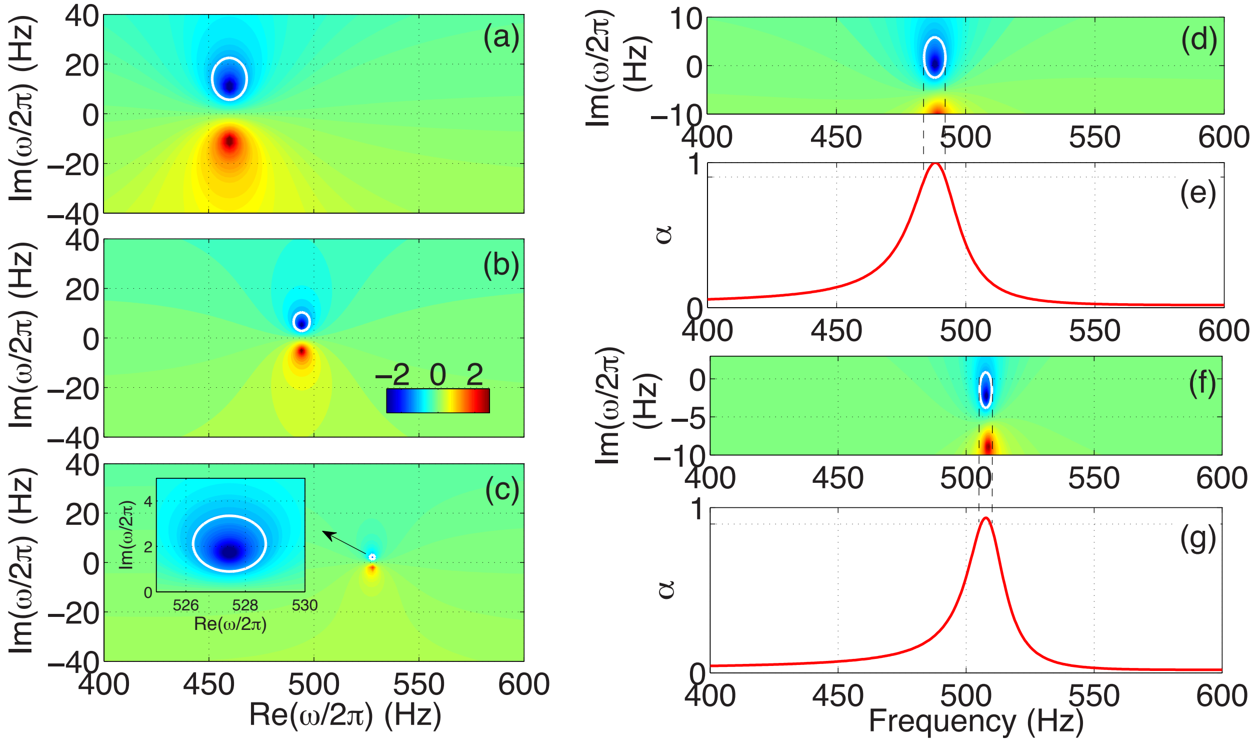

FIG. 2. (a)-(c) Analysis of the reflection coefficient [$\log(|r|)$] in the complex frequency plane in the lossless case for the configurations with L_R = 10, 8.3 and 7 cm respectively. (d) Lossy case for L_R = 8.3 cm. Continuous white line shows the isoline for the value $\alpha = 0.9$. (e) Absorption coefficient in the real frequency axis for the case L_R = 8.3 cm. Horizontal dashed line represents $\alpha = 0.9$. (f) Lossy case for L_R = 7.5 cm. Continuous white line shows the isoline for the value $\alpha = 0.9$. (g) Absorption coefficient in the real frequency axis for the case L_R = 7.5 cm. Horizontal dashed line represents $\alpha = 0.9$.

and the near PA absorption are not observable.

When the inherent losses are introduced into the system, the zeros and the poles are down-shifted, thus this breaks the symmetry of positions with respect to the real frequency axis, allowing the crossing of both the trajectory and the isoline with the real frequency axis. Figures 2(d) and (e) respectively show the reflection coefficient in the complex frequency plane of the configuration with L_R = 8.3 cm and the absorption coefficient in the real frequency axis, taking into account the inherent losses (we notice that the horizontal dashed line in Fig. 2(e) represents $\alpha = 0.9$). We can see now that the zero is on the real frequency axis, accomplishing the PA condition and representing a configuration for observable PA.

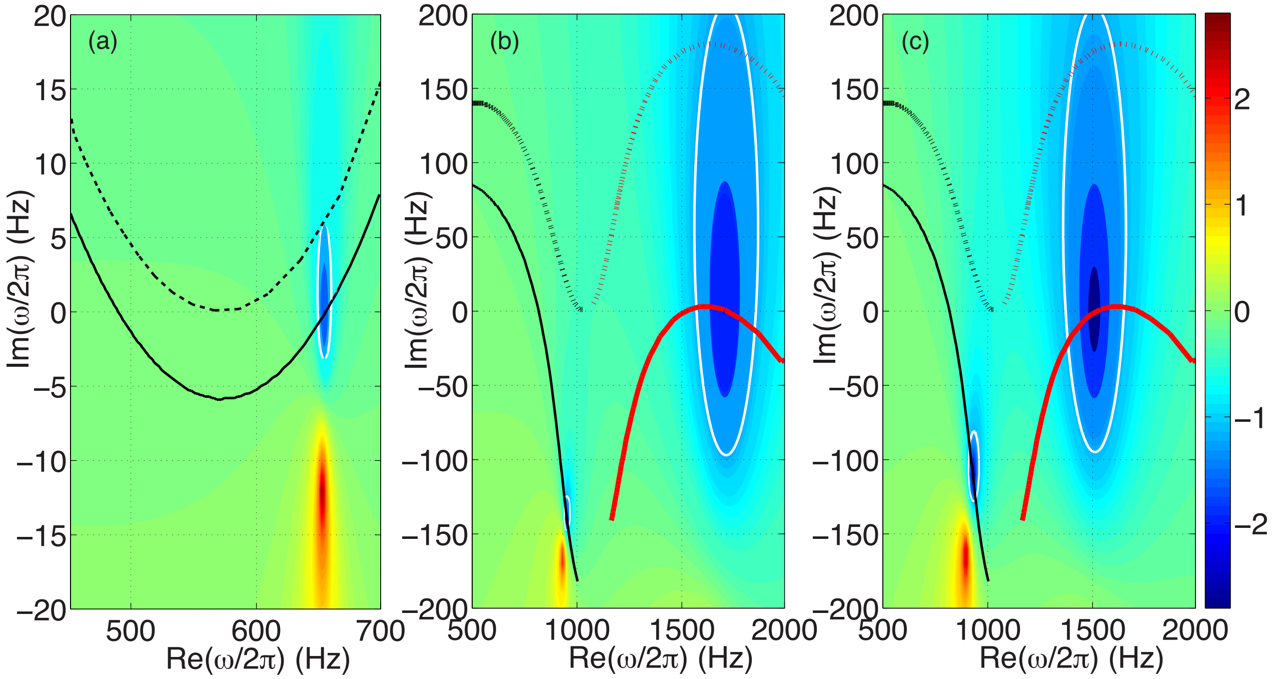

FIG. 3. Reflection coefficient ($\log(|r|)$) evaluated in the complex frequency plane for: (a) the SRS configuration with $(L_R, f) = (3.9 \text{ cm}, 647 \text{ Hz})$, black dashed (continuous) line shows the trajectory of the zero evaluated in the lossless (lossy) case; (b) the SMRS configuration with $(L, f) = (1 \text{ cm}, 1703 \text{ Hz})$ and (c) $(L, f) = (1.37 \text{ cm}, 1508 \text{ Hz})$. Black and red dashed (continuous) lines show the trajectories of the zero for the first and second modes of the system evaluated in the lossless (lossy) case. White continuous line represent the isoline $\alpha = 0.9$.

Moreover, the iso-line $\alpha = 0.9$ crosses the real frequency axis generating the nearly PA around the PA condition, as shown in Fig. 2(e)

In Figs. 2(f)-(e) we respectively plot the reflection coefficient in the complex frequency plane of the configuration with $L_R = 7.5$ cm and the absorption coefficient in the real frequency axis, taking into account the inherent losses. In this case, the zero is off-axis but the isoline $\alpha = 0.9$ crosses the real frequency axis. Then, the PA is not observable, but a peak with nearly PA is observable.

A. Additional complex maps for the SRS and SMRS configurations

Figure 3 shows the complex frequency maps for the rest of the PA configurations shown in the article. Figure 3(a) shows the SRS configuration with $(L_R, f) = (3.9 \text{ cm}, 647 \text{ Hz})$, where black dashed (continuous) line shows the trajectory of the zero evaluated in the lossless (lossy) case. Figure 3(b) shows the SMRS configuration with $(L, f) = (1 \text{ cm}, 1703 \text{ Hz})$ and Fig. 3(c) with $(L, f) = (1.37 \text{ cm}, 1508 \text{ Hz})$. Black and red dashed (continuous) lines show the trajectories of the zero for the first and second modes of the system evaluated in the lossless (lossy) case.

III. PARAMETRIC STUDY: DEPENDENCE OF THE ABSORPTION COEFFICIENT ON THE GEOMETRICAL PARAMETERS

If the lossy material of the plate is fixed, the system is characterized in a 4D space, $\alpha = \alpha(R, h, L; f)$. We will show that, for the material used in this work and with the geometry shown in the manuscript, the results for the cavity length $L = 2.71$ cm are optimal in terms of broadband and perfect absorption.

Upper panels of Fig. 4 show the reflection coefficient for the lossless case in the complex frequency plane ($h_p = 4h, 2h, h, h/2$ and $h/4$ with $h = 3.5$ mm the value of the thickness analyzed in our work) with a closed cavity length of $L = 2.71$ cm. In all cases the plate has the same radius as that of the waveguide, $R = R_p = 2.2$ cm. We can see that, for the same cavity length, when the thickness of the plate is either $h_p < h$ or $h_p > 2h$, the zeros and poles are very close to the real axis, meaning that these resonances present small leakage. Then, due to the fact that our material has large losses, these poles will be down-shifted far away from the real frequency axis, so the effect on the absorption coefficient will be weak. In the case of $h < h_p < 2h$ we see that in the lossless case the zeros have similar imaginary components i.e., they present almost the same leakage. Particularly, for the case $h_p = h$, there are two zeros which have almost the same leakage at close real frequencies. In our case, the losses of the system compensate this leakage, it means, down-shift the zeros very close to the real frequency axis, and then produce two overlapped peaks with absorption near to one.

Bottom panels of Fig. 4 show the dependence of the absorption coefficient on the length

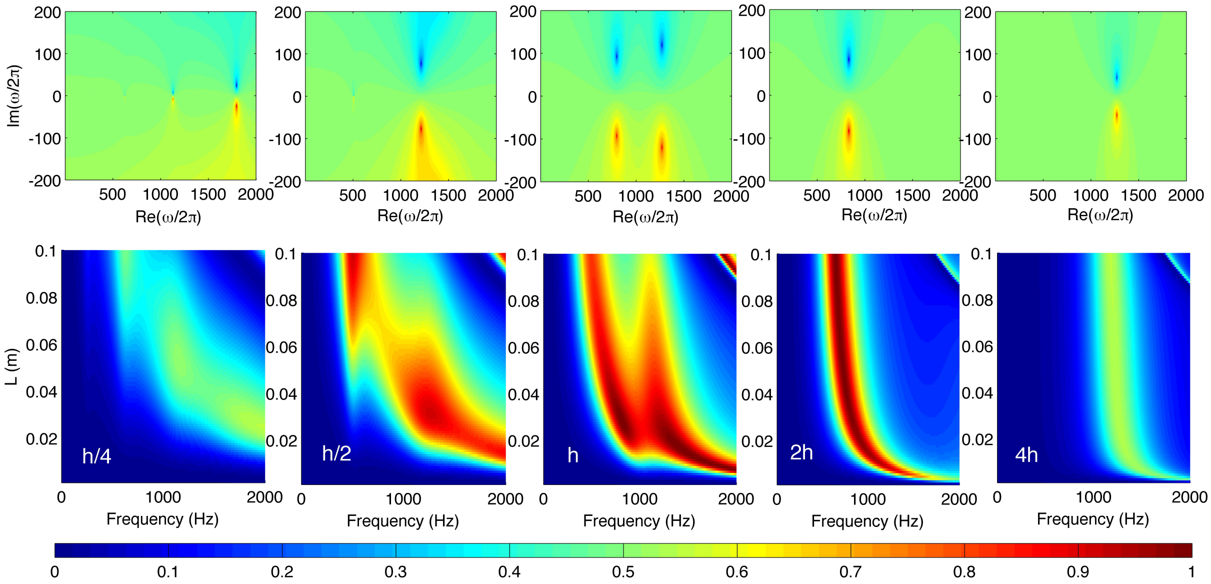

FIG. 4. (Color online) From left to right: results for a plate thickness of $h/4, h/2, h, 2h, 4h$ (with $h = 3.5$ mm which is the thickness of the analyzed system in the manuscript). The radius of the plate is 2.2 cm. Upper panels: lossless analysis of the $\log(|r|)$ in complex frequency plane for the SMRS system. Bottom panels: Dependence of the absorption coefficient on the backing cavity length, L.

cavity for the different systems. As previously mentioned for the cases $h_p < h$ or $h_p > 2h$, we can see that the absorption coefficient never reaches $\alpha = 1$, while for the case $h < h_p < 2h$, the absorption coefficient reaches the value $\alpha = 1$ and, particularly, for $h_p = h$ the two peaks overlap in such a way that we obtain high absorption (one of them showing Perfect Absorption).

IV. EFFECT OF INHERENT LOSSES ON THE CRITICAL COUPLING

The theoretical model analyzed in this work allows the study of the dependence of the absorption properties on the amount of inherent losses of the resonant plate. Therefore, we compare here three cases: (i) the small amount of only viscothermal losses ($\chi = 50$ kg m^3 s$^{1/2}$, $\eta = 0$ Pa s), (ii) the small amount of only viscoelastic losses ($\chi = 0$ kg m^3 s$^{1/2}$, $\eta = 0.75$ Pa s) and (iii) the real case analyzed in this work ($\chi = 350$ kg m^3 s$^{1/2}$, $\eta = 7$ Pa s).

We study now the effect of these inherent losses on PA condition. In Figs. 5(a), 5(b) and

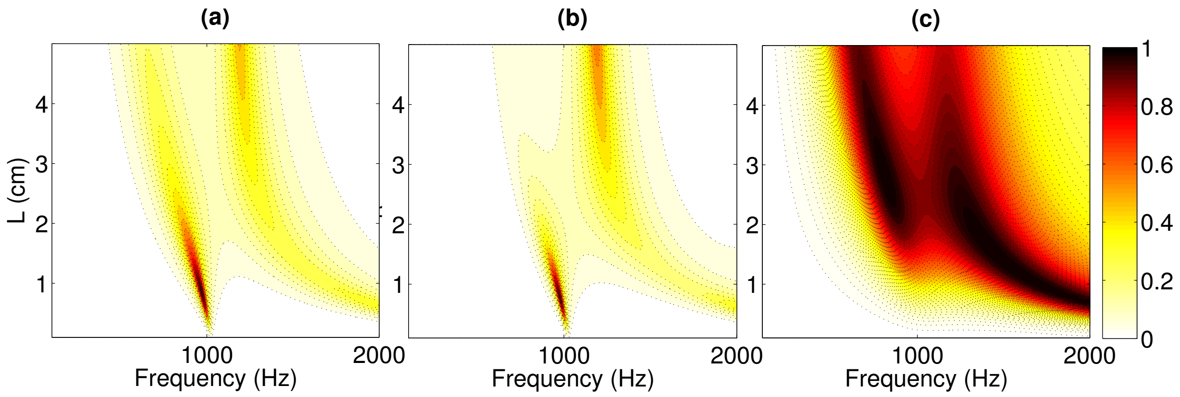

FIG. 5. (Color online) Theoretical study of the effect of inherent losses on the critical coupling. (a), (b) and (c) show the evolution of the absorption coefficient of the plate for three cases: (a) small viscothermal losses ($\chi = 50$ kg m^3 s$^{1/2}$, $\eta = 0$ Pa s), (b) small viscoelastic losses ($\chi = 0$ kg m^3 s$^{1/2}$, $\eta = 0.75$ Pa s) and (c) the case with the real situation ($\chi = 350$ kg m^3 s$^{1/2}$, $\eta = 7$ Pa s). The length of the baking cavity, L, changes from $L = 1$ mm to $L = 5$ cm

5(c) we analyze the evolution of the absorption coefficient on the frequency f, and on the length of the backing cavity L, for the three cases analyzed in this supplementary material, [(i), (ii), (iii)], respectively. We represent the absorption coefficients for the cases of a cavity length varying from $L = 0.1$ cm to $L = 5$ cm.

To understand the effect of the inherent losses on the PA condition for the SMRS, we should keep in mind the trajectories of the zero of the reflection coefficient for the first and second resonant modes for the lossless case (shown in Fig. 3 of the manuscript). For the cases with small amount of inherent losses, shown in Figs. 5(a) and (b), the zeros that cross the real frequency axis represent modes with small leakage rate. For these cases, only the first mode, crosses the real frequency axis in the range of interest of in this work. Due to the small leakage rate of this resonances, the quality factor of the resonance is high, explaining the narrow character of this absorption peak. When the inherent losses are included, the down-shift of the lossless trajectories is bigger than in the previous case. We analyze in Fig. 5(c) the case analyzed in the article, allowing the crossing with the real frequency axis for the first and second modes in three different configurations. Oppositely to the case with small losses, the quality factor of these modes is low ($Q^{-1} = 1$), explaining the broadband character of the absorption peaks, giving rise to the possibility to overlap the several peaks to produce broadband absorption with some of the resonant modes activating

the PA condition.

---

[1] B. H. Song and J. S. Bolton, J. Acoust. Soc. Am. **107**, 1131 (2000).

[2] G. Theocharis, O. Richoux, V. Romero-García, A. Merkel, and V. Tournat, New. J. Phys. **16**, 093017 (2014).

[3] F. Bongard, H. Lissek, and J. Mosig, Phys. Rev. B **82**, 094306 (2010).

[4] V. Pagneux, *Trapped Modes and Edge Resonances in Acoustics and Elasticity, in Dynamic Localization Phenomena in Elasticity, Acoustics and Electromagnetism* (CISM International Centre for Mechanical Sciences, Vol. 547, pp. 181-223, Springer, Vienna, 2013).
